# Simple Method for Evaluation of Planum Temporale Pyramidal Neurons Shrinkage in Postmortem Tissue of Alzheimer Disease Patients

**DOI:** 10.1155/2014/607171

**Published:** 2014-02-11

**Authors:** Martina Kutová, Jana Mrzílková, Denisa Kirdajová, Daniela Řípová, Petr Zach

**Affiliations:** ^1^Institute of Anatomy, Third Medical Faculty, Charles University at Prague, Ruská 87, Prague 10, 100 00 Praha, Czech Republic; ^2^Prague Psychiatric Center, Ústavní 91, Prague 8, 181 03 Praha, Czech Republic

## Abstract

We measured the length of the pyramidal neurons in the cortical layer III in four subregions of the planum temporale (transitions into superior temporal gyrus, Heschl's gyrus, insular cortex, and Sylvian fissure) in control group and Alzheimer disease patients. Our hypothesis was that overall length of the pyramidal neurons would be smaller in the Alzheimer disease group compared to controls and also there would be right-left asymmetry in both the control and Alzheimer disease groups. We found pyramidal neuron length asymmetry only in controls—in the transition into the Sylvian fissure—and the rest of the subregions in the control group and Alzheimer disease patients did not show size difference. However, control-Alzheimer disease group pyramidal neuron length comparison revealed (a) no length difference in superior temporal gyrus transition area, (b) reversal of asymmetry in the insular transition area with left insular transition significantly shorter in the Alzheimer disease group compared to the control group, (c) both right and left Heschl's gyrus transitions significantly shorter in the Alzheimer disease group compared to the control group, and (d) right Sylvian fissure transition significantly shorter in the Alzheimer disease group compared to the control group. This neuronal length measurement method could supplement already existing neuropathological criteria for postmortem Alzheimer disease diagnostics.

## 1. Introduction

Neurodegeneration in Alzheimer disease (AD) affects structures of the temporal lobe (MTA) and in particular the supratemporal plane of the temporal lobe—planum temporale (PT) (for review see [1]). PT is mostly a heteromodal auditory association region whose asymmetry is established by 31 weeks of gestation. Functionally, it is involved in auditory and spatial objects processing, auditory-motor integration, music pitch and tune recognition, and sound localization functions [2], and its structural and functional organization was recently revised. Neuroanatomical and neurophysiological evidence suggest subdivision of the PT into anterior and posterior parts. The anterior part belongs to the auditory cortex proper supporting spatially related but not spatially specific functions like stream segregation. The posterior part is not part of the auditory cortex and it supports sensory-motor integration of the vocal tract actions [3]. At the gross anatomy level, the PT exhibits leftward asymmetry (approx. in 60% of the population) of its length and/or area. In clinical populations was observed reversal of this asymmetry in dyslexia and a loss of asymmetry combined with an increase in right PT size in schizophrenia [4]. Anatomical changes of the PT in dementia were not extensively studied. Microanatomical changes were found in the cortex of the PT in patients with AD in the form of minicolumn thinning, although this finding was not well correlated with decline in cognitive functions [5]. Age associated minicolumn thinning in normally aged people was found in the medial temporal gyrus and temporal lobe association cortex but not in Heschl's gyrus [6]. Our previous anatomical study of PT in AD revealed overall volume and cortical width decrease in AD compared to controls together with rightward asymmetry reversal in AD [7]. We were interested in whether this gross anatomical finding could be correlated with the histological level. If so, PT could be another brain region (besides the hippocampus, prefrontal cortex, gyrus collateralis, locus coeruleus of the brain stem, and cerebellum) that is included in neuropathological examinations in postmortem AD diagnostics.

In our present study, we measured shrinkage of layer III pyramidal neurons in four different parts of the PT (transition to superior temporal gyrus, transition to Heschl's gyri, and transition into insula and posterior part of Sylvian fissure) on the left and right side. Cytoarchitectonically, the first three parts are classified as auditory parakoniocortices (internal: PaAi, external: PaAe, and caudodorsal: PaA) with prominent granularity in layer IV and sparse layer V. The fourth part is nonauditory temporal-parietal area—Tpt—occupying the posterior side of the PT with a weak layer IV and prominent layer V. The reasons for selecting layer III pyramidal neurons for shrinkage measurement were anatomical abnormalities of the PT were observed particularly in the upper cortical layers I–III of the Tpt part of PT in the left hemisphere in schizophrenics [8], axons of layer III pyramidal neurons do not project outside the cortex and their apical dendrites are most prominent, and finally relative homogeneity in the presence of layer III pyramidal neurons across the PT compared to other layers.

We expected more prominent pyramidal neuron size loss in AD compared to controls and also a change towards rightward asymmetry in AD compared to controls.

## 2. Materials and Methods

This study uses the same brain PT tissue samples that were conserved from our past anatomical study on a new volumetric method of AD postmortem diagnosis. Clinical diagnosis of AD as well as postmortem verification on autoptic tissue, demographic data, method of extraction of PT samples from postmortem brains, brain tissue fixation, control patient characterization, and other data were fully described in our previous study [7]. Out of 84 formerly studied postmortem brains, we selected 10 AD PT and 7 control PT (age and sex matched, similar severity of neuropathological findings in the case of AD).

**Figure 1:**
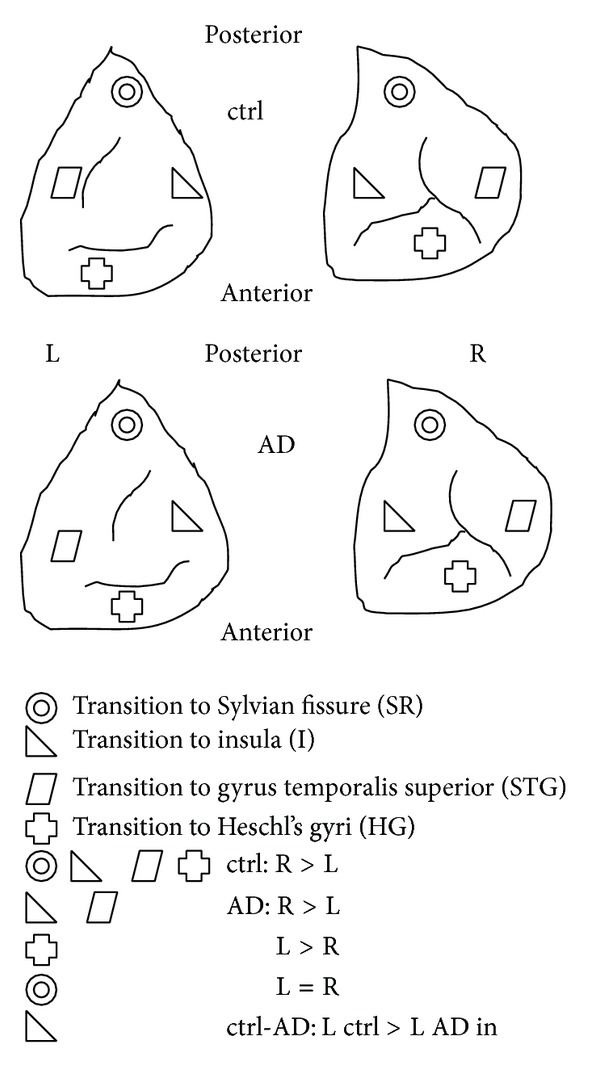
Example of the PT sample from ctrl and AD group (above view, left and right control and AD). Four symbols indicate four positions within the PT where brain tissue was collected and where pyramidal neurons in the cortical layer 3 were measured (STG, HG, I, and SR). Ctrl = control group, AD = Alzheimer disease group, L = left, and R = right. Equations in the lower half of the picture show asymmetries within ctrl, AD, and between ctrl and AD.

Out of each PT embedded in paraplast four samples were cut at the following anatomical localizations: (1) at the lateral border as the superolateral margin of the superior temporal gyrus (STG), (2) at the medial border of the PT at the point of transition into the insular cortex (I), (3) at the ventral border at the point of transition into one of the Heschl's gyri (HG), and (4) at the dorsal border at the point where the Sylvian fissure terminates or bifurcates into posterior descending ramus (SR) (Figure 1) (for more detailed regional description see [1]). All samples were cut on a standard rotary microtome Leica (30 *μ*m slice thickness) with the knife positioned vertically to the cortical surface. Slices were mounted on poly-lysine coated glass slides and stained according to standard Nissl cresylviolet staining. Pyramidal neurons from layer III of the cortex (where most disorganization was previously reported [6]) were studied on a Leica DMLB microscope with 10x magnification and images were digitally captured. Digital images were transferred onto a standard PC and opened in freeware image analysis program Image J 1.47 (http://rsb.info.nih.gov/ij/download.html) with implemented stereological tools. Image J was first calibrated using a calibration slide micrometer (OptixCam, USA). Length of the pyramidal neurons was measured manually by PC mouse pointer after software magnification of the images. Briefly, a thin line was drawn by mouse from the apical dendrite towards the midst of the base of the pyramidal neuron where basilary dendrites were emerging. Measured data in micrometers were stored in the Excel database. All length measurements were done blind to the status of the AD or controls using double blind labeling system of the glass slides.

**Figure 2:**
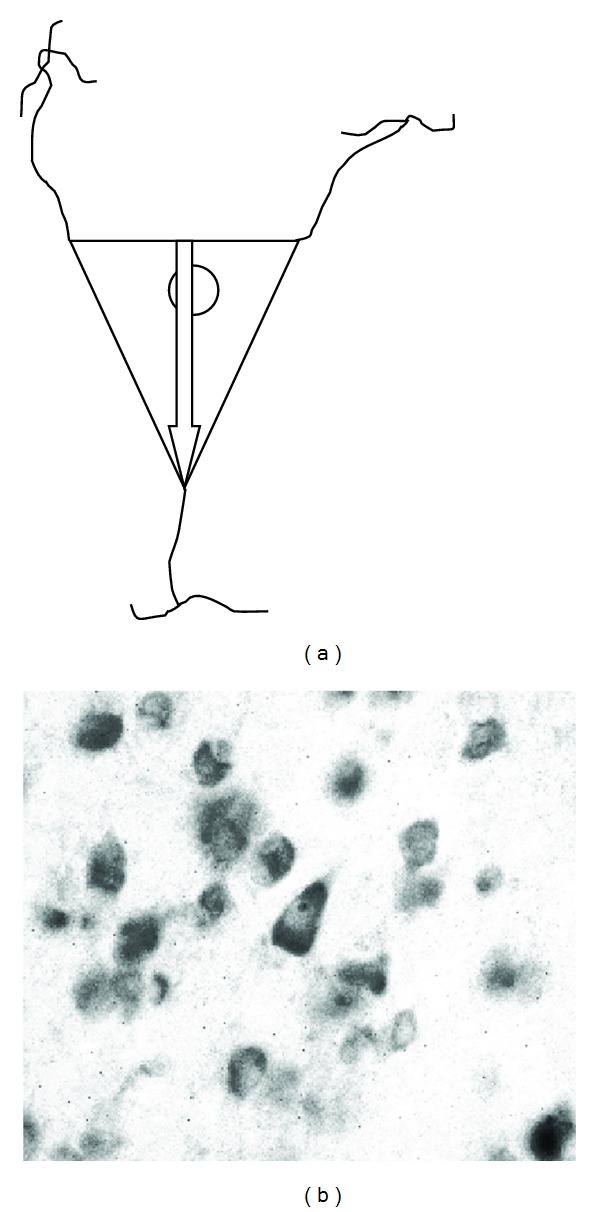
Shape of selected pyramidal neurons of the PT cortical layer III from STG, HG, I, and SR subregions. (a) Base of the arrow represents the starting and tip of the arrow represents the terminating point of the length of the cell body measurement. Tip of the arrow is pointing to the apical dendrite. Only neurons having at the same time visible apical dendrite, both basilary dendrites emerging from the cell body and full contour of the cytoplasm membrane with nucleus inside, were selected into measurement. The ratio of the size of cell, basilary dendrites, and apical dendrite is only for illustration in this picture. (b) Typical pyramidal neuron selected for measurement (in the middle), Nissl stained section, 30 *μ*m thickness, magnification 400x.

Measured neurons were selected according to their pyramidal shape and horizontal position. Their length was estimated by our modification of the nucleator method [9]. Instead of measuring the length of 3 isotropic random lines from a nucleolus to the cell periphery, we measured a straight line connecting the apical dendrite with the point on the opposing cell membrane in between two emerging basilary dendrites. Criteria for inclusion of pyramidal neuron into measurement were position in the cortical layer III, triangular shape of the cell body and clearly visible whole cytoplasmic membrane and clearly visible apical dendrite leaving the cell body and at the opposite side of two or more emerging basilary dendrites. This way we believe we avoided anisotropy in the shape and orientation of the sampled neurons as mentioned in [9]. Such criteria should guarantee horizontal position of the pyramidal neurons relatively to the camera view (see Figure 2). Always five randomly selected neurons were measured in all of the samples (STG, I, HG, SR) on the left (L) and right (R) sides so we measured 680 neurons in total.

The data were exported from Excel into Statistica 10 software. ANOVA with repeated measures was selected for control-AD group differences and Newman-Keuls post hoc analysis for statistical significance [10]. *t*-test for dependent samples was used to evaluate R/L asymmetries within control and AD group. Statistical significance was accepted at *P* = 0.05.

## 3. Results

**Figure 3:**
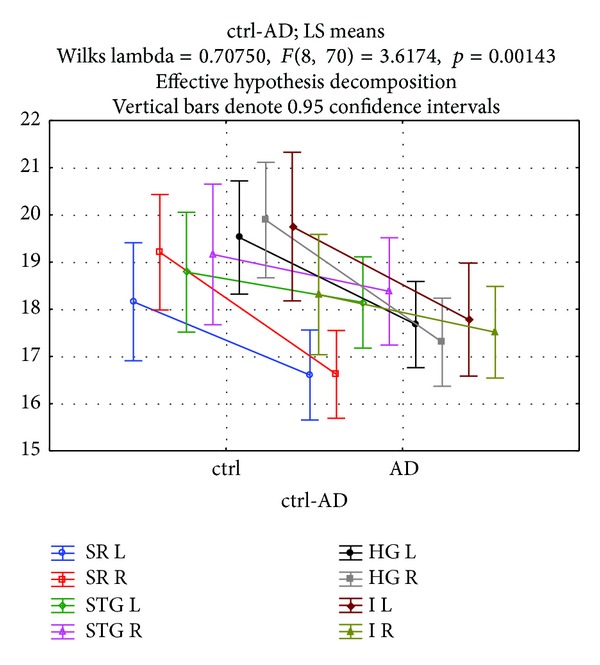
Lenght of pyramidal neurons in cortical layer 3 of the PT subregions in control and AD groups for R and L sides. Length of the cell bodies is in *μ*m. Abbreviations: R: right, L: left, STG: PT transition to superior temporal gyrus, HG: PT transition to Heschl's gyri, I: PT transition to insula and SR: PT transition to Sylvian fissure, ctrl: control group, and AD: Alzheimer disease group.

**Figure 4:**
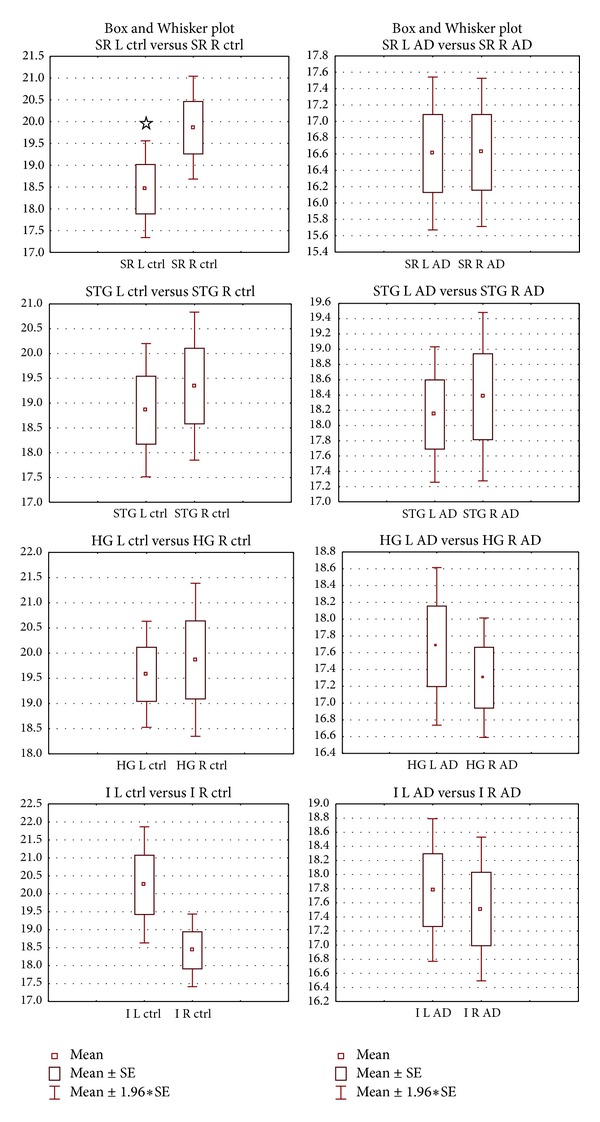
Left-right asymmetries in the length of the layer III pyramidal neurons in the PT of the control (left) and AD (right) groups. *Y*-axis data are in *μ*m. Data are presented as means ± SE. Abbreviations: SR: transition to Sylvian fissure, STG: transition to gyrus temporalis superior, HG: transition to Heschl's gyri, I: transition to insula, L: left side, and R: right side. Significance between left and right side estimated by dependent samples *t*-test at *P* < 0.05 is marked by ☆.

In all subregions (STG, I, HG, SR), length of pyramidal neurons was higher in the control group compared to the AD group. Dependent *t*-test showed that within the control group, there was R > L length for all subregions except for I (L > R) (Figure 4). Within the AD group, there was R > L for STG, reversal of asymmetry (L > R) for HG, decrease of L > R asymmetry (but still L > R) for I, and R = L for SR. Comparison of control-AD group by ANOVA with repeated measures yielded mixed results: no significance in STG, L AD < L control in I, both L and R AD < control in HG and R AD < R control in SR (Figure 3). In terms of within groups asymmetry, we found significance only in the SR of the control group (R > L).

### 3.1. STG

R > L in both control (w/o significance) and AD groups (w/o significance). No significant difference exists between control and AD groups on both sides.

### 3.2. I

L > R in control (w/o significance) and AD groups (almost equal, *P* = 0.78). L is significantly shorter in AD compared to control group (*P* = 0.04).

### 3.3. HG

R > L in the control group (w/o significance) and L > R in the AD group (w/o significance). Both R and L are significantly shorter in the AD group compared to control group (R; *P* = 0.001, L; *P* = 0.01).

### 3.4. SR

R > L in the control group (*P* = 0.04) and R = L in the AD group (*P* = 0.98). R is significantly shorter in AD compared to control group (*P* = 0.003).

## 4. Discussion

We measured length of PT cortical layer III pyramidal neurons in 7 controls and 10 AD patients on postmortem brain tissue. As mentioned in our previous study [7], PT tissue samples of AD patients and controls were embedded in paraplast in 2009, so we expected unavoidable tissue shrinkage. We presume similar tissue shrinkage due to the same paraplast embedding protocol for all samples. Also, all paraplast embedded PT tissue samples were stored in the same dry boxes at room temperature until sectioning. Interestingly, although we did not sort out pyramidal neurons according to their cell body size, our results ranged typically from 15 to 22 *μ*m regardless of AD or control groups. If there would be a significant difference in shrinkage between samples, then the neurons would exhibit greater size variability. This is the reason we did not calculate tissue shrinkage corrections [11].

Our findings show that only one subregion of PT, SR, had larger cell bodies (R > L) and only in the control group. This part of the PT is a nonauditory cortex involved in sensory-motor integration of vocal tract actions [3]. In the AD group in all subregions, we did not find significant R/L asymmetry. One region (I) showed that L > R, and one region (SR) is almost equal to R = L. This is in contrast with a study reporting that L > R in controls (cells in the auditory or speech areas—but only magnopyramidal cells accounting for 10% of the cells sorted for binomial analysis for hemisphere lateralization out of great size variability of total pyramidal neurons were measured [12]). Our observation of the lack of R/L asymmetry in the AD group could be seen as a decrease of rightward dominance that is normally present in controls. Similarly, well-known rightward decrease in hippocampal volume in AD leads to call-down of normally present asymmetry in controls [13]. Therefore, the posterior part of the PT can be another area specifically lesioned by AD, in addition to the gyrus collateralis, entorhinal cortex, hippocampus, and brain stem. Due to participation of Tpt in the vocal processing streams, its decreased size layer III pyramidal neurons could contribute to vocalization problems in patients with AD in combination with declarative memory impairment. This would however be valid only for population with rightward lateralized auditory and speech centers since we did not find a decrease in neuronal size on the left side. Reduced neuronal size in layer III of the PT was also observed in bipolar disorder [14] but not in schizophrenia and major depressive disorder, and significance was lost when adjusted for six layerwise comparisons. Also reduction of somal volume of the layer III pyramidal neurons of the temporal association cortex was described in schizophrenia [15], so our finding may not be limited to the extent of the PT but rather with overlaps to neighbouring regions. Our observed reversal of asymmetry in the HG region (R > L to L > R) is opposite to reversals of PT asymmetry observed in schizophrenia (R > L) [1].

Layer III: external pyramidal layer of the cortex predominantly contains small- and medium-sized pyramidal neurons and also nonpyramidal cells. Pyramidal cells are principal targets of interhemispheric corticocortical afferents and also a primary source of corticocortical efferents. Rightward lateralized decrease of their size in AD may not be restricted only to the layer III, and that could be a topic of further studies.

## 5. Conclusion

We present neurohistological methodology of measuring length changes in layer III pyramidal neurons in the PT (especially in its subparts of I, SR, and HG) in patients with progressed Alzheimer disease. The data from our previous study [7] showed right/left laterality in the cortical thickness, area and volume of the PT in controls, and change into left/right laterality in the AD group. In the present study, we found similar right/left laterality in the control group and a decrease, disappearance, or reversal of this asymmetry in the AD group. Therefore, gross changes in the PT in AD may be at least partially attributed to neurohistological changes in the layer III pyramidal neurons. We suggest that this method of pyramidal neuron length measurement could be used in addition to existing clinical neuropathological evaluations of AD in hospitals.

## References

[1] Shapleske J, Rossell SL, Woodruff PWR, David AS (1999). The planum temporale: a systematic, quantitative review of its structural, functional and clinical significance. *Brain Research Reviews*.

[2] Ahveninen J, Kopco N, Jääskeläinen IP (2014). Psychophysics and neuronal bases of sound localization in humans. *Hearing Research*.

[3] Hickok G, Saberi K, Poeppel D (2012). Redefining the functional organization of the planum temporale region: space, objects, and sensory-motor integration. *The Human Auditory Cortex*.

[4] Ratnanather JT, Poynton CB, Pisano DV (2013). Morphometry of superior temporal gyrus and planum temporale in schizophrenia and psychotic bipolar disorder. *Schizophrenia Research*.

[5] Chance SA, Clover L, Cousijn H, Currah L, Pettingill R, Esiri MM (2011). Microanatomical correlates of cognitive ability and decline: normal ageing, MCI, and Alzheimer’s disease. *Cerebral Cortex*.

[6] Chance SA, Casanova MF, Switala AE, Crow TJ, Esiri MM (2006). Minicolumn thinning in temporal lobe association cortex but not primary auditory cortex in normal human ageing. *Acta Neuropathologica*.

[7] Zach P, Krištofiková Z, Mrzílková J (2009). Planum temporale analysis via a new volumetric method in autoptic brains of demented and psychotic patients. *Current Alzheimer Research*.

[8] Smiley JF, Rosoklija G, Mancevski B, Mann JJ, Dwork AJ, Javitt DC (2009). Altered volume and hemispheric asymmetry of the superficial cortical layers in the schizophrenia planum temporale. *European Journal of Neuroscience*.

[9] Gunderson HJG (1988). The nucleator. *Journal of Microscopy*.

[10] Freedman D, Pisani R, Purves R (2007). *Statistics*.

[11] West MJ (2013). Tissue shrinkage and stereological studies. *Cold Spring Harb Protoc*.

[12] Hutsler JJ (2003). The specialized structure of human language cortex: pyramidal cell size asymmetries within auditory and language-associated regions of the temporal lobes. *Brain and Language*.

[13] Mrzilková J, Zach P, Bartoš A, Tintera J, Řípová D (2012). Volumetric analysis of the pons, cerebellum and hippocampi in patients with Alzheimer's disease. *Dementia and Geriatric Cognitive Disorders*.

[14] Beasley CL, Chana G, Honavar M, Landau S, Everall IP, Cotter D (2005). Evidence for altered neuronal organisation within the planum temporale in major psychiatric disorders. *Schizophrenia Research*.

[15] Sweet RA, Pierri JN, Auh S, Sampson AR, Lewis DA (2003). Reduced pyramidal cell somal volume in auditory association cortex of subjects with schizophrenia. *Neuropsychopharmacology*.

